# Splicing Players Are Differently Expressed in Sporadic Amyotrophic Lateral Sclerosis Molecular Clusters and Brain Regions

**DOI:** 10.3390/cells9010159

**Published:** 2020-01-08

**Authors:** Valentina La Cognata, Giulia Gentile, Eleonora Aronica, Sebastiano Cavallaro

**Affiliations:** 1 Institute for Biomedical Research and Innovation, National Research Council, 95126 Catania, Italy; 2 Department of (Neuro)Pathology, Amsterdam UMC, University of Amsterdam, Amsterdam Neuroscience, Meibergdreef 9, 1105 Amsterdam, The Netherlands

**Keywords:** amyotrophic lateral sclerosis, spliceosome, splicing factors, SALS molecular subtypes, motor cortex, spinal cord, tissue-specific program, hub-bottlenecks, non hub-bottlenecks, *YBX1*

## Abstract

Splicing is a tightly orchestrated process by which the brain produces protein diversity over time and space. While this process specializes and diversifies neurons, its deregulation may be responsible for their selective degeneration. In amyotrophic lateral sclerosis (ALS), splicing defects have been investigated at the singular gene level without considering the higher-order level, involving the entire splicing machinery. In this study, we analyzed the complete spectrum (396) of genes encoding splicing factors in the motor cortex (41) and spinal cord (40) samples from control and sporadic ALS (SALS) patients. A substantial number of genes (184) displayed significant expression changes in tissue types or disease states, were implicated in distinct splicing complexes and showed different topological hierarchical roles based on protein–protein interactions. The deregulation of one of these splicing factors has a central topological role, i.e., the transcription factor YBX1, which might also have an impact on stress granule formation, a pathological marker associated with ALS.

## 1. Introduction

Amyotrophic lateral sclerosis (ALS) is a progressive and fatal disease of motor function, characterized by the selective degeneration of upper and lower motor neurons. Alternative splicing plays an important role in neuronal differentiation, and the dysregulation of RNA processing and splicing is a key contributor to ALS pathogenesis [1,2]. Indeed, not only do a vast number of alternative splicing defects involve ALS associated genes, but several of their encoded products participate in RNA processing and splicing (FUS, TDP-43, C9ORF72, ATXN2, TAF15, hnRNPA1, hnRNPA2B1, MATR3, EWSR1, SETX) [1,2].

RNA splicing is one of the major drivers of eukaryotic RNA metabolism, allowing the removal of introns and the joining of exons from precursor mRNA in a constitutive or alternative manner. Nearly all human multi-exon genes undergo splicing, but this process is particularly widespread in the brain, where splicing programs allow for cellular protein diversity [3,4,5]. Although RNA splicing represents an extremely economical means for increasing protein diversity, in the terms of both time and space, finely tuning the genomic information to meet the unique needs of each neuron, it adds an additional layer of complexity and vulnerability [6]. In fact, while this process specializes and diversifies neurons, its deregulation may be responsible for their selective degeneration.

To date, splicing defects in ALS have been investigated at the singular gene level without considering the higher-order level involving the splicing machinery [2]. Indeed, RNA splicing is mediated by the spliceosome, a dynamic macromolecular machinery comprised of more than 100 individual components [7]. Unlike many enzymes, the spliceosome does not have a preformed catalytic center. Instead, it is constructed de novo on the pre-mRNA in a stepwise manner for each round of splicing and undergoes multiple rearrangements to configure the catalytic core [8]. The major spliceosome machine is composed of five small nuclear ribonucleoprotein complexes, (snRNPs), U1, U2, U4, U5, and U6, each containing a cognate U-rich noncoding small nuclear RNA (snRNA). A small subset of unusual introns containing non-consensus termini are processed by a much less abundant spliceosomal machine (minor spliceosome) which contains the U5 snRNP and analogs of the remaining snRNPs (U11, U12, U4atac, and U6atac) [8,9,10,11,12]. Following transcription, snRNAs are exported through the nuclear pore complex (NPC) into the cytoplasm where snRNPs maturate inside a large assemblysome (the survival motor neuron (SMN) complex); core snRNPs are then imported into the nucleus, assembled in Cajal bodies and are ready to be recruited by the nascent pre-mRNA for splicing [8,13]. The basepairing of snRNPs with pre-mRNA relies on a series of conserved regulatory sequence elements (the splice donor site, the splice acceptor site, the branch site, and the polypyrimidine tract) [14]. Additional layers of regulation are introduced by some auxiliary motifs (exon and intron splicing enhancers or silencers) that may stimulate exon inclusion or inhibit splicing with the mediation of RNA-binding proteins (RBPs), mainly of which belong to the families of serine/arginine-rich splicing factor (SRSF) proteins and heterogeneous nuclear ribonucleoproteins (hnRNPs), acting as splicing enhancers or repressors, respectively [15,16]. For a complete overview of splicing machinery, intermediated complexes and auxiliary regulatory factors, readers are invited to refer to some complete reviews [8,17,18,19].

To investigate the role of the spliceosome in ALS, here we analyzed the entire spectrum (396) of genes encoding splicing factors in the motor cortex and spinal cord samples from control and sporadic ALS (SALS) patients. These genes encode both the core spliceosomal components involved in constitutive splicing, as well as hnRNPs (usually splice site inhibitors), SRSFs (usually splice site activators) and additional splicing factor regulators. A substantial number of genes displayed significant expression changes in tissue types or disease states and showed different topological hierarchical roles based on protein–protein network interactions. One of these, the transcription factor YBX1, might have an impact on stress granule formation, pathological markers associated with ALS.

## 2. Materials and Methods

### 2.1. Data Source and Gene Selection

For this study, we referred to two transcriptome data sets, both available at ArrayExpress (http://www.ebi.ac.uk/arrayexpress/) with the accession numbers E-MTAB-2325 (https://www.ebi.ac.uk/arrayexpress/experiments/E-MTAB-2325/) (previously described as reported in [20]) and E-MTAB-8635 (https://www.ebi.ac.uk/arrayexpress/experiments/E-MTAB-8635/) (new submission). These data sets consisted of the mRNA expression profiles of 41,059 genes from 41 motor cortex samples and 40 spinal cord samples of SALS and control subjects (Table 1) hybridized onto 4 × 44K Whole Human Genome Oligo expression microarrays (Agilent Technologies, Turin, Italy). A detailed description of the study design, subject characteristics and experimental procedures was reported in the original publication [20]. In order to identify the common or differential genomic changes between the spinal cords and motor cortexes belonging to the same cohort of patients, the sample preparation, RNA extraction and microarray processing of the spinal cord tissues obtained from patients of the same cohort were performed as previously described [20]. The transcriptomic data obtained from the spinal cord were combined with data from the motor cortex to create a complete dataset of 81 whole transcriptomic observations, as listed in Table 1. Once obtained, the raw intensity values were thresholded to 1, log2-transformed, normalized, and baselined to the median of all samples by using GeneSpring GX (Agilent Technologies).

### 2.2. Gene List Filtering and Differential Expression Analysis

The whole-genome expression data were filtered to include probes targeting genes involved in the splicing core machinery or the auxiliary regulatory factors. In particular, an a priori splicing gene list was generated using query keywords in the Gene Ontology (GO) database (http://www.geneontology.org/). Overall, 396 genes were selected from the following GOs: Spliceosomal complex (GO:0005681), Regulation of mRNA splicing, via spliceosome (GO:0048024), mRNA splicing, via spliceosome (GO: 0000398). Moreover, a number of additional genes (n = 15) were filtered from SPLICE AID-F (http://srv00.recas.ba.infn.it/SpliceAidF/) [21] and included in the gene panel. A full list of the a priori splicing selected genes is reported in Supplementary Data S1.

The statistical analysis of the filtered microarray data was performed with the GeneSpring GX v13.1 software package (Agilent Technologies), applying a two-way analysis of variance (ANOVA) followed by Benjamini–Hochberg false discovery rate (FDR) correction to identify the differentially expressed genes (DEGs) and minimize false-positive cases. A two-way ANOVA test was used, since it offers the possibility to identify genes (i) significantly deregulated across the first variable (disease state: Control or SALS subtypes), (ii) significantly deregulated across the second variable (Tissue types: motor cortex or spinal cord), and (iii) showing a significant interaction between the two variables. The list of probes that were statistically significant, together with their expression levels in the motor cortex and spinal cord of the control and SALS patients, is reported in Supplementary Data S2.

Probes with an adjusted *p*-value < 0.05 and an FC value greater than ±2 were used as candidate deregulated genes for further exploration in 7 pairwise correlations relevant to the study’s aim (Cortex SALS1 vs. Cortex CTRL; Cortex SALS2 vs. Cortex CTRL; Spinal Cord SALS1 vs. Spinal Cord CTRL; Spinal Cord SALS2 vs. Spinal Cord CTRL; Cortex SALS1 vs. Spinal Cord SALS1; Cortex SALS2 vs. Spinal Cord SALS2; Cortex CTRL vs. Spinal Cord CTRL). For further analysis, the probes were converted into the corresponding genes. When two or more probes targeted the same gene, the mean expression level was considered to address the up- or down-regulation.

To visualize the results, Venn diagrams were generated by using the Interactivenn web tool (http://www.interactivenn.net/) [22].

### 2.3. Functional Characterization of Differentially Expressed Splicing Factors

To understand which splicing complexes were implicated in ALS, the differential expressed genes were enriched by their cellular component gene ontology (GO) and analyzed statistically by applying Fisher’s Exact test with the use of an overrepresentation test in the web-based, open-access PANTHER database, using the microarray gene list as a reference (see Supplementary Data S3). The *p*-value were adjusted by using Bonferroni correction for multiple testing and *p* < 0.05 was set as the significance threshold.

### 2.4. Protein–Protein Interaction Network Analysis

To evaluate the potential protein–protein interaction (PPI) in the motor cortex, we generated two human tissue-specific networks using NetworkAnalyst v.3.0 [23] and the data belonging to the human tissue-specific db DifferentialNet [24], using as input data two DEG lists in SALS1 vs. control cortex and SALS2 vs. control cortex. The former contained 79 DEGs, while the latter was composed of 176 DEGs. The two gene lists were used as the input data in NetworkAnalyst v.3.0, together with their logFC values, in order to indicate the direction of the expression change, and duplicates were filtered using the log FC mean. Using the NetworkAnalyst tool, the input data were matched with their corresponding human brain cortex-specific proteins using the differential protein–protein interactions human tissue-specific database, i.e., DifferentialNet db, filtered by a percentile threshold of 15%, and were then used to generate tissue-specific first-order PPI networks.

The same procedure was applied to the spinal-cord SALS1 and SALS2 DEG lists, made up, respectively, by 8 and 5 DEGs. Their data were used to create spinal-cord specific first order PPI networks filtered by a percentile threshold of 15%.

The data referring to the descriptive statistics of the tissue-specific networks for both SALS clusters were obtained using the PHStat2 add-in for excel and are shown in the Supplementary Data S5.

To calculate the cut-off for hub-bottlenecks and nonhub-bottlenecks, we referred to the method described in [25], identifying the nodes with a degree greater than or equal to the sum of the mean and twice the standard deviation (mean + 2*S.D. of the degree distribution) as hub-bottlenecks. While nonhub-bottleneck screening was performed by sorting the node betweenness distribution in descending order and selecting the nodes with values lying in the top 50% of the betweenness distribution. The nodes ranging from those with a degree less than the degree cut-off for hub-bottlenecks, directly connected to at least two hub-bottlenecks, and with a betweenness value, were selected as nonhub-bottlenecks.

Venn diagrams for both the hub-bottleneck and nonhub-bottleneck categories in the tissue-specific networks for both ALS clusters were obtained using the Interactivenn web tool [22] (http://www.interactivenn.net/).

A literature-based network was obtained using the Genomatix Pathways System (GePS, www.genomatix.de) using as the input list the tissue-specific DEGs involved in cytoplasmic stress granule formation and belonging to GO:0010494 (*CASC3*, *FMR1*, *DDX1*, *PABPC1*, *YBX1*, *HNRNPK*, *MBNL1*, *CIRBP*, *TARDBP,* and *RBPMS*) in *Homo sapiens*. The gene expression log FC values were added using the Metadata import option.

## 3. Results and Discussion

### 3.1. Identification of Differentially Expressed Genes in Tissue Types and Disease States

**Figure 1:**
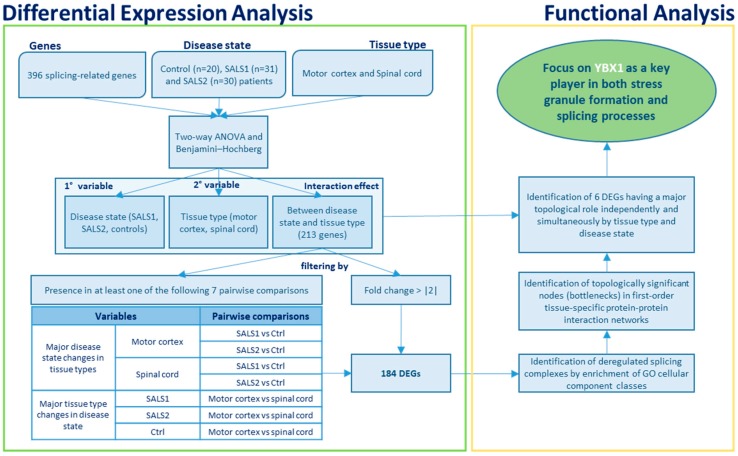
The flow chart illustrates the analysis methods used in the present study to characterize the differentially expressed genes encoding splicing factors (left green box) and investigate their functional significance (right orange box).

To identify the complete repertoire of the splicing components differentially regulated in SALS, we investigated the expression profiles of 396 splicing factors in the motor cortex (n = 41) and spinal cord (n = 40) samples from control and SALS patients. In two previous studies, we were able to successfully discriminate controls from SALS patients and distinguish these latter in two greatly divergent SALS subtypes (SALS1 and SALS2) by using unsupervised hierarchical clustering [20,26]. Each of these subtypes were characterized by specific transcriptomic alterations associated with divergent deregulated genes and pathways, allowing a molecular taxonomy of patients. The existence of distinct SALS molecular clusters has also been recently confirmed in an RNA-seq study [27]. Since the union of SALS subgroups into a single disease entity would conceal the great existing variability in ALS, in the present study, patients and tissues (both cortex and spinal cord) were analyzed taking into account the previously demonstrated separation. Therefore, we performed the differential gene expression analysis by comparing the controls to each of the two SALS subtypes previously characterized. Figure 1 shows globally the analysis methods used in the present study to characterize the differentially expressed genes encoding splicing factors and investigate their functional significance.

**Figure 2:**
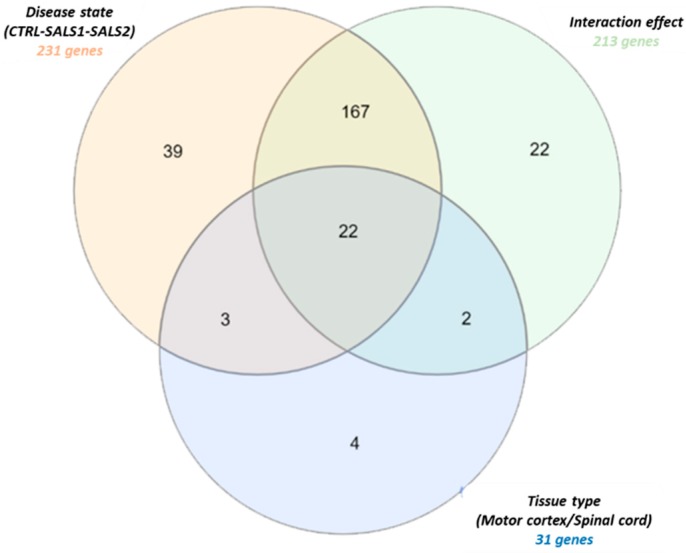
Venn diagram of differentially expressed splicing factor genes in tissue districts (motor cortex and spinal cord) of control and SALS subtype patients. Detailed information for the lists of gene probes is provided in Supplementary Data S2.

A two-way analysis of variance (ANOVA) with multiple test correction (Benjamini–Hochberg’s FDR test) was applied to measure the gene expression changes influenced by each of the two variables, tissue type (motor cortex and spinal cord) and disease state (control, SALS1, and SALS2), independently or simultaneously. Through this analysis, 259 genes out of 396 were identified as having a significant differential expression (*p* < 0.05) with respect to the tissue type, disease state or both (Supplementary Data S2). The Venn diagram in Figure 2 displays the number of statistically significant genes influenced independently and/or simultaneously by the two variables. A total of 31 genes were differentially expressed across the tissue types, 231 genes were significantly deregulated across the disease states, whereas 213 genes showed an interaction effect and changed in the two tissue types along with the disease states (Figure 2). Twenty-two genes were deregulated in the disease state, tissue type and also in their interaction effect (Figure 2).

**Figure 3:**
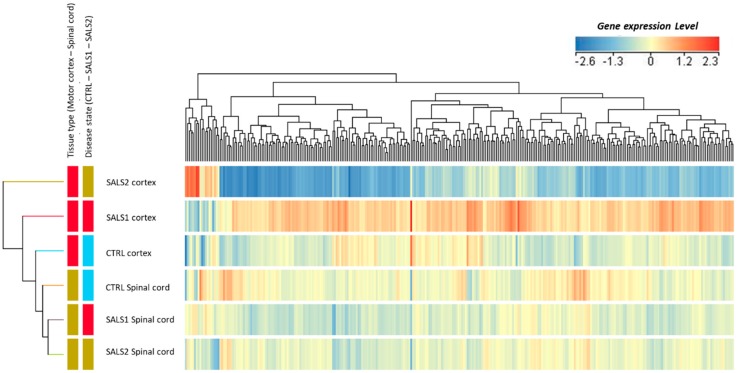
Supervised hierarchical clustering was used to visualize the gene expression changes in differentially expressed genes (302 entities) in the motor cortexes and spinal cords of control and ALS (SALS1 and SALS2 subtypes) patients. As shown in the color bar, red indicates up-regulation, and blue down-regulation.

Figure 3 shows the supervised cluster of gene entities (n = 302 probes corresponding to 213 genes) deregulated in the two tissue types, along with the disease state (interaction effect, Figure 2). These transcriptomic profiles confirm the great divergence between SALS1 and SALS2 motor cortex samples, as previously reported in [20], and a less pronounced difference in spinal cord samples.

Next, we focused on genes (n = 184) out of 213 showing major expression changes (fold change > |2|) in different pairwise correlations that were relevant to the study’s aim (Figure 1 and Supplementary Data S3).

**Figure 4:**
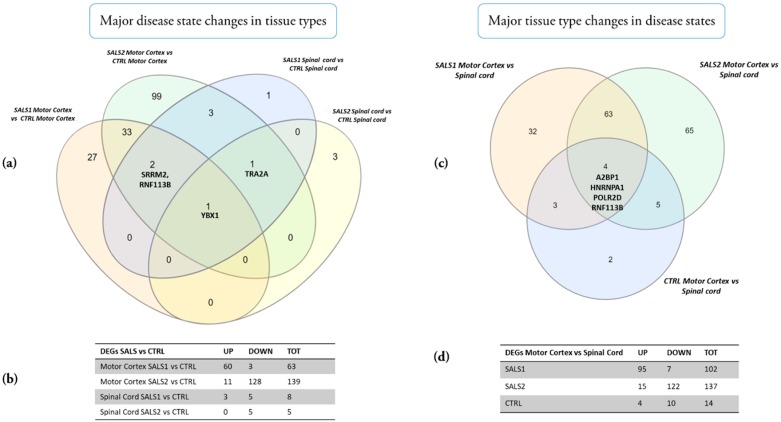
Venn diagrams of the total number of deregulated genes in SALS subtypes versus controls (**a**) and in motor cortex versus spinal cord (**c**). Tables show the numbers of up and down-regulated genes in SALS subtypes versus controls (**b**) and in motor cortex versus spinal cord (**d**), respectively.

The results reveal that major changes according to the disease state in the different tissue types occurred, particularly in the SALS2 motor cortex vs. the control cortex (DEGs = 139, the majority of which were down-regulated), followed by the SALS1 cortex vs. the control (DEGs = 63, the majority were up-regulated), while minor expression change events occurred in the spinal cord of both SALS1 and SALS2 compared to the controls (DEGs = 8 and 5, respectively) (Figure 4 and Supplementary Data S3). *YBX1* was the only gene commonly deregulated in the comparison of tissue types; *TRA2A* was down-regulated in both SALS1 and SALS2 spinal cords vs. the controls and up-regulated in the SALS2 cortex compared to the controls. *SRRM2* and *RNF113B* were differentially expressed in both SALS1 and SALS2 cortexes, and SALS1 spinal cords (Figure 4a,b).

The major changes according to tissue type in the different disease states are shown in Figure 4c,d and Supplementary Data S3. A small number of genes (n = 14) were differentially expressed in the motor cortex vs. spinal cord of control patients, whereas a significant deregulation was observed in SALS1 (DEGs = 102, mostly up-regulated in the cortex) and SALS2 (DEGs = 137, mostly down-regulated in the cortex). Four genes were deregulated in all tissue type comparisons (*A2BP1*, *HNRNPA1*, *POLR2D*, *RNF113B*).

The differential expression of genes encoding the subset of splicing factors in motor cortex of SALS1 and SALS2 patients was compared with their corresponding whole transcriptome analysis previously published [20]. This comparison showed a complete overlap in terms of genes and their regulation for splicing-related DEGs and, at the same time, highlighted the contribution of the gene set focused analysis in putting in light the deregulation of other splicing-related genes not previously assessed (Supplementary Data S3). Moreover, a further comparison between DEGs and their copy number variants (CNVs), previously screened using a neurological exon-custom aCGH [28], was also performed, highlighting the *SMN1* recurrent loss (penetrance > 10%) in SALS2 patients (chr5:70220768-70249769) with a concordance between gene expression deregulation and CNV (Supplementary Data S3).

### 3.2. Deregulated Splicing Complexes

DEGs encode factors that are involved in different splicing complexes. functional enrichment analysis of their GOs allowed us to group these factors into different splicing complexes and identify which of these were significantly deregulated in specific disease states and tissue types (Table 2 and Supplementary Data S3). Functional enrichment analysis was performed starting from DEGs in each of the seven pairwise comparisons. To reduce redundancy and simplify their comprehension, the most significant overrepresented GOs are summarized in Table 2 and are listed in Supplementary Data S3bis. In the following paragraphs, we will discuss the expression changes of these splicing factors by focusing on the main functional groups (splicing complexes), highlighting common features and specific differences.

#### 3.2.1. Spliceosomal Complex Core

##### U1 snRNP

Among the five canonical ribonucleoprotein complexes, U1 snRNP is the most studied in ALS. Genes composing U1 snRNP were globally downregulated in SALS2 motor cortexes compared to the control, and with a divergent expression trend in SALS1 and SALS2 tissue type comparisons. U1 is constituted of 18 components and is well known to interact with the ALS-associated FET (*FUS*, *EWSR1*, *TAF15*) proteins, with *MATR3*, as well as with the SMN complex, required for intranuclear gem formation (Gemini of coiled bodies) and snRNP biogenesis [29,30,31,32]. Mutations in *FUS* determined a poor interaction with U1, causing a mis-localization to the cytoplasm and a global loss of splicing activity [33,34]. In addition, the knockdown of any of the U1 snRNP-specific proteins resulted in a dramatic loss of SMN-containing gems and in motor axon truncations in zebrafish models [34].

##### U2 snRNP

Several DEGs in the motor cortexes of both disease states and in SALS tissue type comparisons were overrepresented in U2-coordinated spliceosomal complexes (Table 2). Enriched GOs included the U2-type pre-spliceosome (formed by association of U1 snRNP to the 5′ splice site and U2 snRNP to the branch point sequence), the precatalytic spliceosome (determined by the recruitment of the preassembled U4/U6.U5 tri-snRNP), the catalytic step 1 (or GT-AG activated spliceosome) and the catalytic step 2 spliceosome (an intermediate complex in which the first catalytic cleavage has already occurred).

The disruption of U2 snRNP localization, activity and function was described in ALS patients carrying the expanded *C9ORF72.* This latter encodes aberrant dipeptide repeat (DPR) proteins, which prevent spliceosome assembly through interaction with U2 particles [35], blocking them in the cytoplasm far away from splicing sites and producing mis-spliced cassette exons in *C9ORF72* patient brains [35].

A number of U2 subunit DEGs (*SF3B5*, *SNRNP200, SF3B1, MFAP1, PABPC1)* interact with, or are genetic modulators of, ALS pathogenic genes, including *FUS*, *TDP*-*43*, and *VCP* [30,36,37,38,39], while others (*SYF2*) have been associated with neuronal apoptosis [40]. Noteworthy, a consistent dysregulation of the *SRRM2* splicing factor was previously described in substantia nigra, amygdala and the peripheral blood of parkinsonian patients, drawing attention to the role of this gene in neurodegeneration [41].

##### U4, U5, U6 snRNPs and tri-snRNPs Complex

U4/U6.U5 tri-snRNP is a 1.5 MDa pre-assembled spliceosomal complex comprising U5 snRNA, extensively base-paired U4/U6 snRNAs and >30 proteins. This tri-snRNP combines with a pre-mRNA substrate bound to U1 and U2 snRNPs and transforms into a catalytically active spliceosome, following extensive compositional and conformational changes triggered by the unwinding of the U4/U6 snRNAs [42].

U6 snRNA expression levels are regulated by TDP-43 and its amount significantly decreased after TDP-43 depletion, leading to neuronal cell death [43]. Two U6 components (*LSM7* and *LSM4)* were involved in FUS toxicity suppression and exhibited alterations in ALS fibroblasts [44,45], while *SNRPN* and *SNRPC* expression changed following FUS depletion and toxic *C9ORF72*-derived DPR exposure [35,46].

Other interesting splicing factors include *PRPF6* and *PRPF8*, which were previously identified as hub genes in a network/pathway-based analysis conducted to investigate differential pathogenic mechanisms in sporadic ALS [47]. Interestingly, *PRPF6* is located inside an ALS critical genetic region, carrying SNPs significantly associated with ALS pathogenesis in Japanese people [48].

#### 3.2.2. U12-Dependent Minor Splicing Pathway

Despite the majority of overrepresented GOs being related to the U2-dependent splicing pathway, some DEGs are involved in the minor splicing pathway (U12-dependent). The minor counterpart of the splicing machinery regulates the splicing of introns with atypical AT–AC terminal dinucleotides, and has received increasing attention during the last few years as a novel pathomechanistic player in both neurodevelopmental and neurodegenerative diseases [9].

It has been recently suggested as a possible pathomechanism by which mutated FUS inhibits the correct minor splicing pathway. Mutated FUS seems to forms cytoplasmic aggregates, which are able to trap U11 and U12 snRNAs inside [49]. Moreover, a TDP-43 loss-of-function was shown to determine a disturbance of U11/U12 spliceosomes in both tissues affected by ALS and cellular models [50].

*YBX1* (or YB-1) deserves a particular mention, as it is the only gene commonly deregulated in both tissue types and disease states. This gene, a highly conserved transcription factor known to be a potential drug target in cancer therapy [51,52], is likely to have important local implications for motor neuron activity. Its mRNA was recently reported as being abundantly enriched in distal axons of motor neurons compared to the soma compartment, where it may support the local regulation of distal axonal processes [53]. It has also been described as an essential inducer-splicing of the human muscle-specific receptor tyrosine kinase (*MuSK*) gene, a postsynaptic transmembrane molecule that mediates the clustering of acetylcholine receptors [54].

#### 3.2.3. Exon–Exon Junction Complex (EJC)

The exon junction complex (EJC) is a protein complex deposited onto mRNAs following splicing, and is able to both stably bind mRNAs and function as an anchor for further processing steps. Indeed, EJC acts as a central node of post-transcriptional gene expression networks and regulates mRNAs’ cellular localization, translation and non-sense mediated decay (NMD) [55].

The dysregulation of EJC was found in both SALS1 and SALS2 motor cortexes versus controls, and in SALS tissue type comparisons, but with opposite expression trends (Table 2). Interestingly, one of the central EJC effectors (*UPF1)* is a key-player in ALS and has been proposed as a novel therapeutic target for treatment. Its expression preserves forelimb function in a rat model of TDP-43-induced motor paralysis [56] and reduces toxicity in primary mammalian neurons expressing mutant FUS or TDP-43 [57,58], as well as efficiently blocking neurotoxicity caused by *C9ORF72* DPR [59,60]. The depletion of the EJC member *SRSF1* inhibits the nuclear exporting of pathological *C9ORF72* transcripts, the production of DPR and alleviates neurotoxicity in several human neuronal models [61,62]. Other EJC constituents (*UPF3B* and *EIF4A3)* were upregulated in ALS cell lines and were shown to interact with FUS and TDP-43 [58,63].

#### 3.2.4. CRD-Mediated mRNA Stability Complex

The CRD-mediated mRNA stability complex is a protein complex that binds to and promotes the stabilization of mRNA molecules containing the coding region instability determinant (CRD). Among the complex members, *HNRNPU* was previously identified as one of five top-ranked RNA binding proteins, showing significant alterations in the motor neurons, cerebellum and spinal cords of ALS patients compared to the control, and co-localizes with cytoplasmic TDP-43 aggregates [64,65]. Moreover, we observed in SALS2 motor cortexes vs. control a down-regulation of *SYNCRIP*, whose overexpression was previously found to ameliorate spinal muscular atrophy pathological phenotypes [66].

#### 3.2.5. RNA Polymerase II, Core Complex

In SALS2 motor cortexes and both SALS tissue type comparisons, we observed the deregulation of genes involved in the RNA polymerase II core complex. Thanks to the association of U1 snRNP with the RNAP II machinery, this transcription potently enhances splicing, and vice versa, by a mechanism of reciprocal coupling [67,68].

RNAP2 is the largest subunit of RNA polymerase II, and its phosphorylation state at the C-terminal domain is orchestrated by FUS. When FUS is missing, RNAP2 accumulates at the transcription start site and causes a shift in mRNA transcripts expression toward early polyadenylation sites [69]. Moreover, defects in FUS and TDP-43 recruitment influence RNAPII termination and determine R-loop (DNA:RNA hybrids) accumulation, leading to elevated DNA damage at transcription terminators [70]. Also, the recently described ALS-associated gene *MATR3* interacts with *POLR2A*, *POLR2B*, and *POLR2C* at the early steps of transcription [71].

#### 3.2.6. Prp19 Complex

The conserved Prp19 complex (Prp19C)—also known as NineTeen Complex (NTC)—acts in several processes for cellular homeostasis maintenance, but its best-characterized function is in splicing. NTC/Prp19C is a non-snRNP splicing complex crucial that plays multiple roles: it associates with the assembling spliceosome during or after the dissociation of the U4 snRNP, stabilizes the U5/U6 snRNP in the activated spliceosomal complex (B-act) and remains linked with the spliceosome during the second step of splicing [72]. The DEGs involved in the Prp19 complex were specifically deregulated in SALS2 cortexes vs. control cortexes and in SALS2 tissue type comparison (Table 2).

A Prp19 complex member (*HSPA8)* encodes a constitutive heat shock protein which is able to associate with TDP-43 [36,73]. The level of this protein was found reduced at the neuromuscular junctions in both mutated TDP-43 mice and human models, as well as in motor neurons carrying the *C9ORF72* repeat expansion [74].

#### 3.2.7. SMN–Sm Protein Complex

The SMN–Sm protein complex is a stable multiprotein complex present in all eukaryotic cells that surveys the correct identity of the target RNAs and mediates the biogenesis of snRNPs. It is composed of SMN (the survival motor neuron) protein, at least six additional proteins named Gemins2–7, which associate with both Sm proteins and snRNAs [75,76].

*SMN1*, the core of the complex, has received great attention in the study of ALS, especially because it is located in a genomic region that frequently undergoes copy number abnormalities [77]. A number of studies have demonstrated that high or low *SMN1* copy numbers increased the risk of developing ALS, although data in the literature are conflicting (all reviewed in [77]). Decreased levels of SMN protein cause a reduction of the chaperone-like activity following SOD1-mediated toxicity, and thus contribute to free radical injury and oxidative stress [78]. On the contrary, increased SMN protein levels protect motor neurons from stress- or ALS mutation-induced death, raising important clinical implications for SMN therapeutics in motor neuronal diseases [79].

#### 3.2.8. Supraspliceosomal Complex

The supraspliceosome is a huge (21 MDa) and highly dynamic RNP machine composed of four active spliceosomes connected to each other by the pre-mRNA [80,81]. This multi-task regulator harbors all five spliceosomal U snRNPs at all splicing stages, and additional pre-mRNA processing components, such as the 5′-end and 3′-end processing factors, and the RNA editing enzymes *ADAR1* and *ADAR2* [80]. ADARs, in particular, catalyzes the hydrolytic deamination of adenosine to inosine in double-stranded RNA (dsRNA), and this process is referred to as A-to-I RNA editing. ADAR isoform down-regulation was previously observed in neurons carrying C9ORF72 mutations and in TDP43-deficient cell lines; moreover these proteins are incorporated into RNA-rich nuclear foci and are targeted directly by TDP43 [82].

### 3.3. Tissue-Specific Networks of Spliceosome-Related Gene Products

To investigate the functional relevance of splicing-related proteins and their direct interactors in both tissues, we generated first-order PPI networks using DEGs which were known to be specific for those tissues, and expanding them to their direct interactions, which were also tissue-specific. After that, we investigated their hierarchical role through the identification of the topological significance of their nodes, as described in the following.

Network topological properties have been described in the literature in terms of functional significance, making a difference between a) highly connected nodes (hubs), and b) nodes which are highly passed through as the shortest paths (bottlenecks). While the former are characterized by a high degree of connection, the latter are identified by high betweenness values, and, in turn, can be divided into nonhub-bottlenecks and hub-bottlenecks, depending on their degree of connection. Since in particular cases of PPI networks with indirect edges, such as signaling transduction and permanent interactions (i.e., protein complexes), betweenness tend to be more essential than degree [83], we analyzed first-order tissue-specific networks of spliceosome-related gene products, looking for bottlenecks categories with high and low degrees of connection. In fact, both categories have their importance, not only in highlighting network centrality (hub-bottlenecks), which could disrupt the network if removed, but also in finding peripheral proteins mediating different pathways, connecting different complexes and/or being involved in cross-talking (nonhub-bottlenecks), both having key roles.

In the next paragraph, we will illustrate the PPI networks of splicing factors deregulated in SALS subtypes, either in the cortex or in the spinal cord.

#### 3.3.1. Cortex-Specific PPI Networks

**Figure 5:**
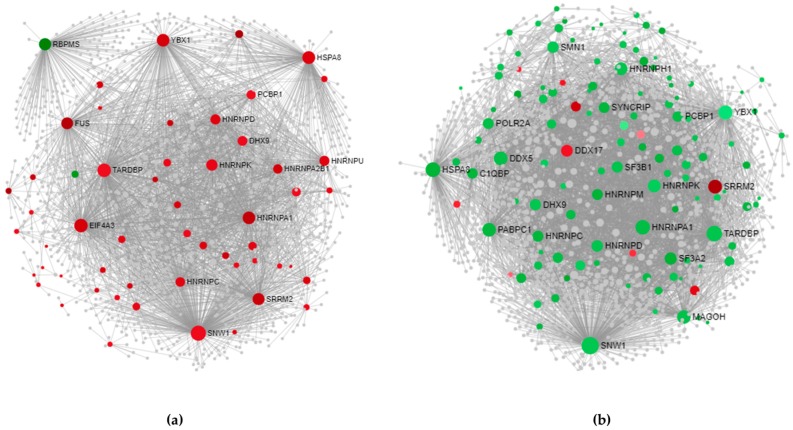
PPI networks in (**a**) SALS1 and (**b**) SALS2 cortexes. To better visualize the generated networks we used the Force Atlas layout algorithm. Sphere size is proportional to the degree of connection, whereas the color (red for up-regulated and green for down-regulated in SALS subtypes) represents the expression logFC value. Details about nodes properties are listed in Supplementary Data S4.

The PPI networks of splicing factors deregulated in SALS1 and SALS2 cortexes and their direct (first-order) interaction partners are illustrated in Figure 5 (network properties and topological significance analysis are described in Supplementary Data S5). Using 55 deregulated tissue-specific genes as seeds for SALS1, we obtained 19 hub- and 338 nonhub-bottlenecks, while 35 hub- and 441 nonhub-bottlenecks were identified for SALS2, starting with 122 seeds (see Supplementary Data S5 for details about hub- and nonhub-bottlenecks). The profound divergence of SALS1 and SALS2 subtypes is evident in Figure 5. Among hub-bottlenecks (depicted in Figure 5 as spheres higher in size), 10 are common to the two SALS subtypes and most of them (*TARDBP*, *HNRNPA1*, *HNRNPC*, *HNRNPD*, *HNRNPK*, *SNWI*, *YBX1*, *HSPA8*, *DHX9* and *PCBP1*) shows an opposite expression trend in the cortex networks, whereas only *SSRM2* is overexpressed in both.

#### 3.3.2. Spinal Cord-Specific PPI Networks

**Figure 6:**
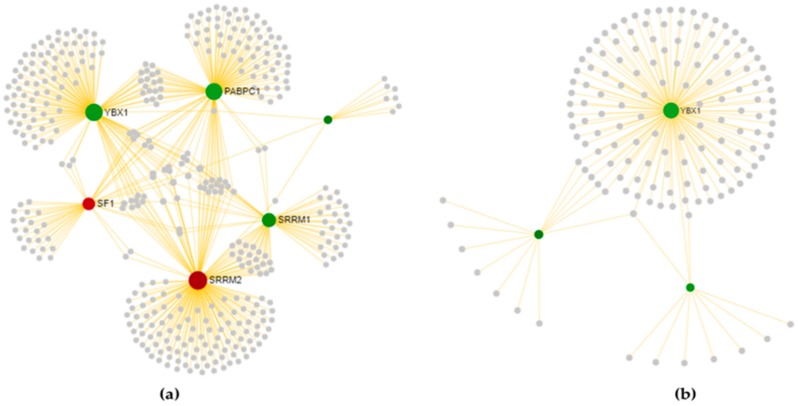
Spinal cord-specific protein–protein interaction (PPI) networks are shown for both (**a**) SALS1 and (**b**) SALS2. To better visualize generated networks, we used the Auto layout algorithm. Sphere size is proportional to the degree of connection, whereas the color (red for up-regulated and green for down-regulated in SALS subtypes) represents the expression logFC value. Details about node properties are listed in Supplementary Data S4.

The PPI networks of splicing factors that are deregulated in SALS1 and SALS2 spinal cords and their direct (first-order) interaction partners are illustrated in Figure 6 (network properties and topological significance analysis are described in Supplementary Data S5). According to DEGs data, spinal cord-specific first-order PPI networks (Figure 6) show a less characterized distinction between SALS1 and SALS2 when compared to those obtained in the cortex (Figure 5). Using six deregulated tissue-specific genes as seeds for SALS1, we obtained five hub- and 92 nonhub-bottlenecks, while only one hub-bottleneck was identified for SALS2 starting with three seeds (see Supplementary Data S5 for details about hub- and nonhub-bottlenecks). As shown in Figure 6, only one hub-bottleneck is common to both disease subtypes, *YBX1*, with a down-regulation of its transcript in both, whereas *SSRM2* appears to be a SALS1-specific hub-bottleneck with an up-regulation of its transcript.

#### 3.3.3. The Most Relevant Splicing-Related Genes

Among the 22 most relevant splicing-related genes (deregulated independently and simultaneously by the two variables, disease state, and tissue type), ten had a relevant topological role (hub- or non-hub-bottleneck) in at least one of the four disease state pairwise comparisons, either in the motor cortex or the spinal cord (see Supplementary Data S5): *YBX1*, *HNRNPU*, *HNRNPC*, *RBM3*, *PABPC1*, *DNAJC8, HNRNPA3, HNRNPUL1, SNRPN,* and *YTHDC1.* Interestingly, three of these (*HNRNPUL1, SNRPN,* and *YTHDC1)* have been recently reported as differentially expressed in peripheral blood sample transcription profiling in a huge heterogeneous ALS cohort (not only sporadic cases), but their up or down regulation was not specified [84]. Moreover, recent evidence has shown that *YBX1*, *HNRNPC*, *PABPC1*, *HNRNPA3*, and *YTHDC1* RNAs bound to TDP-43 protein in human SH-SY5Y neuroblastoma cells using eCLIP-seq [27].

**Figure 7:**
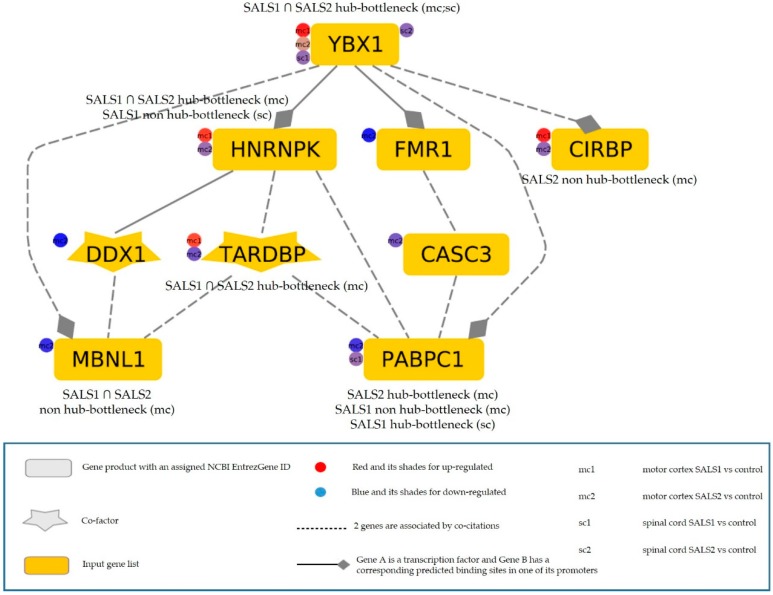
Literature-based network of tissue-specific deregulated genes involved in stress granule formations generated by the Genomatix Pathways System (GePS). The hierarchical layout has been used to highlight the main direction or information flow of the network. The legend below reports information about elements in figure.

YBX1 deserves a particular mention, as not only is it differentially expressed in all four disease state comparisons, but is also known to participate in several steps of the spliceosomal machinery, playing a central topological role as a hub-bottleneck. Recent evidences highlighted the role of YBX1 in regulating protein recruitment for the formation of stress granules [85], which represents a pathological marker associated with multiple neurodegenerative disorders [86]. The ALS-associated proteins TDP-43 and FUS are known to aggregate in stress granules and can be released by YBX1, which represents a key source of protein aggregation regulation for assembling/disassembling stress granules in motor neurons [87]. Based on this evidence, we generated a regulatory network of DEGs implicated in cytoplasmic stress granule formation (GO: 0010494: CASC3, FMR1, DDX1, PABPC1, YBX1, HNRNPK, MBNL1, CIRBP, and TARDBP) to investigate the hierarchical flow coordinating stress granule formation. As shown in Figure 7, the hub-bottleneck YBX1 is the top-gene regulating all the tissue-specific DEGs involved in stress granule formation. The majority of these have a topological significance and are deregulated in at least one condition. Interestingly, the transcription rate of five of them (HNRNPK, FMR1, CIRBP, MBNL1, and PABPC1) may be regulated by YBX1, since they own in their promoters the corresponding predicted transcription factor binding site. Given the role of YBX1 in a number of spliceosomal complexes, its deregulation at different genomic levels may not only alter splicing but also influence gene expression levels of proteins recruited in stress granules, causing aberrations in the assembly/disassembly metabolism.

## 4. Conclusions

A number of scientific studies support the role of alternative splicing dysregulation in ALS. Fluctuations in the concentration of core spliceosomal proteins, as well as additional regulatory factors, may alter gene expression and underly the selective neurodegeneration in ALS.

To date, splicing defects in ALS have been investigated at the singular gene level, without considering the higher-order level involving the splicing machinery. In this study, we analyzed the entire spectrum of genes encoding splicing factors in the motor cortex and spinal cord samples from control and SALS patients, previously characterized as two distinct molecular subgroups (SALS1 and SALS2) [20,26]. The data presented show that a substantial number of splicing-related genes displayed significant expression changes according to tissue type and disease state. Overexpression was a trend for the SALS1 group, while down-regulation was a trend for SALS2, although the DEGs were more pronounced for the motor cortex regions than the spinal cord. A number of DEGs in both SALS1 and SALS2 motor cortexes were involved in spliceosomal complexes or step reaction regulation (i.e., U2-type precatalytic and catalytic step reactions, the U4/U6.5 tri-snRNP complex, the U12-type minor spliceosomal pathway, the CRD-mediated mRNA stability complex and the exon–exon junction complex) while other overrepresented GOs were specific to SALS2 (i.e., supraspliceosomal complex, SMN–Sm protein complex, RNA polymerase II core complex). Data integration between gene expression profiling and topological significance (hub-bottlenecks and nonhub bottlenecks) in network analysis aided us to stratify genes based on their deregulation impact, identifying those that are likely to be more relevant in the balance between physiological and pathological status. Deregulation of one of these bottlenecks, including *YBX1,* might impact stress granule formation, a pathological marker associated with ALS.

Despite it not being possible to completely distinguish the causative factors from secondary degenerative changes ongoing in the diseased brain, it is interesting to note that most of the deregulated splicing factors are known to interact with the ALS-causative mendelian genes, thus suggesting that perturbations in a small subset of splicing components could trigger pathological mechanisms and influence motoneuronal degeneration in sporadic cases.

The existence of transcriptional differences separating SALS patients in two (or more) distinct sub-clusters might explain why several compounds showing promising results in preclinical studies failed to translate into successful clinical trials [27,88]. Indeed, the lack of therapeutic progress may mainly due to an insufficient understanding of the complexity and heterogeneity underlying ALS [89,90]. The investigation of splicing deregulation in both the motor cortex and spinal cord samples into molecularly separated disease groups revealed significant alterations that would be hidden considering ALS as a single disease entity. Moreover, elucidating the impact of splicing machinery changes on global RNAs deregulation may provide novel mechanistic insights underlying the selective degeneration of motor neurons.

Future functional and clinical investigation will certainly be necessary to assess the potential role of candidates’ splicing factors in affecting the origin or modifying the progression of the disease. Notably, the recent technological advancements have made possible the development and use of RNA-targeting molecules as potential therapeutic strategies to increase the expression or mediate the reduction of a given RNA [1,91,92]. This kind of approach may be used in the future to specifically target upstream regulators (e.g., splicing factors or RNA-binding proteins), avoiding large RNAs downstream deregulation that could create more extended damage to cellular physiology.

## Figures and Tables

**Table 1:** Number of samples processed for sporadic amyotrophic lateral sclerosis (SALS) patients’ molecular clusters and regions on the central nervous system (CNS).

Molecular Clusters	Motor Cortex	Spinal Cord
SALS1	18	17
SALS2	13	13
CTRL	10	10
TOT	41	40

**Table 2:** Enriched gene ontologies (GOs) of differentially expressed genes (DEGs) in both disease state and tissue types pairwise comparisons.

Enriched GO Cellular Component	Major Disease State Changes in Tissue Types				Major Tissue Type Changes in Disease States		
	SALS1 Motor Cortex vs Control	SALS2 Motor Cortex vs Control	SALS1 Spinal Cord vs Control	SALS2 Spinal Cord vs Control	Ctrl	SALS1	SALS2
U1 snRNP		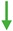 1.55 × 10^9^				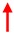 3.70 × 10^7^	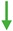 5.18 × 10^8^
A2AF	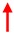 1.24 × 10^2^	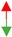 2.87× 10^2^					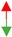 2.82× 10^2^
U2 snRNP		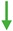 1.59 × 10^9^				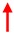 8.44 × 10^9^	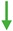 1.38 × 10^9^
U2-type prespliceosome		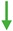 8.35 × 10^10^				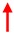 8.33 × 10^6^	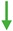 7.23 × 10^10^
U2-type precatalytic spliceosome	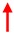 2.84 × 10^6^	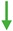 8.57 × 10^28^				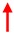 2.80 × 10^15^	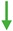 2.89 × 10^26^
U2-type catalytic step 1 spliceosome	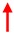 4.21× 10^2^	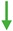 1.46 × 10^4^				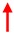 2.65 × 10^3^	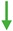 4.01 × 10^6^
U2-type catalytic step 2 spliceosome	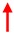 2.29 × 10^7^	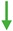 6.89 × 10^13^				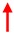 6.47 × 10^11^	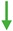 2.09 × 10^11^
U4 snRNP		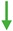 3.36 × 10^3^					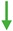 3.22 × 10^3^
U5 snRNP	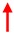 6.80 × 10^7^	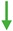 1.54 × 10^8^				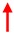 5.47 × 10^6^	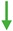 5.55 × 10^7^
U6 snRNP		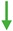 1.38 × 10^6^					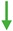 1.26 × 10^6^
U4/U6.U5 tri-snRNP complex	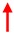 3.39 × 10^4^	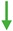 1.61 × 10^17^				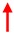 8.01 × 10^11^	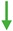 5.64 × 10^16^
U12-type spliceosomal complex	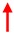 3.75× 10^6^	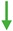 6.35 × 10^9^			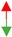 4.30 × 10^2^	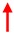 1.09 × 10^6^	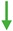 1.81 × 10^7^
Exon-exon junction complex	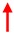 9.41 × 10^5^	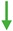 2.07 × 10^9^				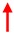 4.60 × 10^7^	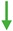 5.09 × 10^11^
CRD-mediated mRNA stability complex	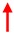 1.90 × 10^4^	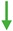 1.02 × 10^3^				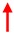 2.70 × 10^2^	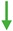 2.35 × 10^5^
RNA polymerase II, core complex		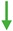 2.84 × 10^10^				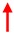 1.65 × 10^4^	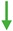 2.45 × 10^10^
Prp19 complex		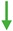 2.44 × 10^4^					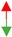 2.38 × 10^4^
SMN-Sm protein complex		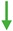 1.90 × 10^5^				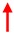 5.93 × 10^3^	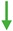 1.64 × 10^5^
SMN complex		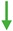 3.31 × 10^3^					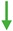 3.18 × 10^3^
pICln-Sm protein complex		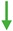 2.68 × 10^5^					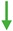 2.39 × 10^5^
Supraspliceosomal complex		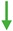 2.84 × 10^4^				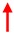 1.64 × 10^4^	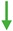 1.43 × 10^2^
Spliceosomal complex				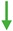 1.11 × 10^4^			
Catalytic step 2 spliceosome			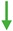 6.15 × 10^3^		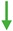 9.80 × 10^3^		
Post-mRNA release spliceosomal complex		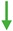 6.29 × 10^3^				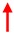 3.74 × 10^3^	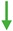 2.34 × 10^4^

For each enriched GO, the Table shows GO cellular component name and the enrichment *p*-value. Red or green arrow indicates if the GO is up or down-regulated.

## Supplementary Materials

The following are available online at https://www.mdpi.com/2073-4409/9/1/159/s1. Data S1: List of splicing-related genes. Data S2: List of significant differentially expressed probes, as resulted from Two Way-ANOVA test and post-hoc correction. Data S3:. List of genes included in the Agilent 4x44K array and used as reference for GO enrichment (sheet 1); up and down deregulated genes in each of the seven pair comparisons (sheet 2); Comparisons between splicing related DEGs, whole transcriptome DEGs and CNVs (sheets 3 and 4). Data S3bis: Over-represented GOs cellular component enrichment of DEGs listed in S3 for each of the seven pairwise comparisons. Data S4: Brain cortex-specific PPI network nodes for both SALS1 and SALS2 clusters (sheet 1 and sheet 2) and spinal cord-specific PPI network nodes (sheet 3 and sheet 4). Data S5: Statistics of degree distribution underlying topological analysis for both brain cortex-specific and spinal cord-specific networks for SALS1 and SALS2 disease clusters.
