# Supplementary material for: Splicing Players Are Differently Expressed in Sporadic Amyotrophic Lateral Sclerosis Molecular Clusters and Brain Regions
Source: Cells. 2020 Jan 8;9(1):159. doi: 10.3390/cells9010159 (PMC7017305; doi:10.3390/cells9010159)
Supplement: Supplementary file 1 [file cells-09-00159-s001.zip › Supplementary_data_S5.docx]

**Data S5 - Topological analysis of brain cortex-specific SALS1 and SALS2 networks**

Data regarding statistics of degree distribution underlying topological analysis for both brain cortex-specific and spinal cord-specific networks for SALS1 and SALS2 disease clusters are shown in tables and figures. In all tissue-specific SALS1 and SALS2 networks, nodes degree distributions are skewed to the right with the mean greater than the median values (Tables 1 and 6). The power-law degree distributions are put in light in figures 1 and 3, highlighting few hubs with a large number of links and the most part of the nodes with few links in both degree distributions. Details about network properties and cut-off used for topological analysis are listed in table 2 and 3 for cortex-specific networks, and table 7 and 8 for spinal cord-specific networks. Hub-bottlenecks and nonhub-bottlenecks are showed in figures 2 (cortex) and 4 (spinal cord), and in table 4 and 5 (cortex), 9 and 10 (spinal cord). Topological role of relevant splicing-related nodes whose transcripts were deregulated in the four disease state pairwise comparisons in different tissue types is highlighted in table 11.

**Table 1.** Descriptive statistics summary concerning degree distributions of brain cortex-specific first-order networks.

| **Descriptive statistics** | **SALS1** | | **SALS2** |
| --- | --- | --- | --- |
| **Mean** | | 3.9 | 5.03 |
| **Median** | | 1 | 1 |
| **Mode** | | 1 | 1 |
| **Minimum** | | 1 | 1 |
| **Maximum** | | 330 | 330 |
| **Range** | | 329 | 329 |
| **Standard Deviation** | | 16.8 | 16.53 |
| **Skewness** | | 11.8 | 10.19 |
| **Count** | | 1479 | 1711 |


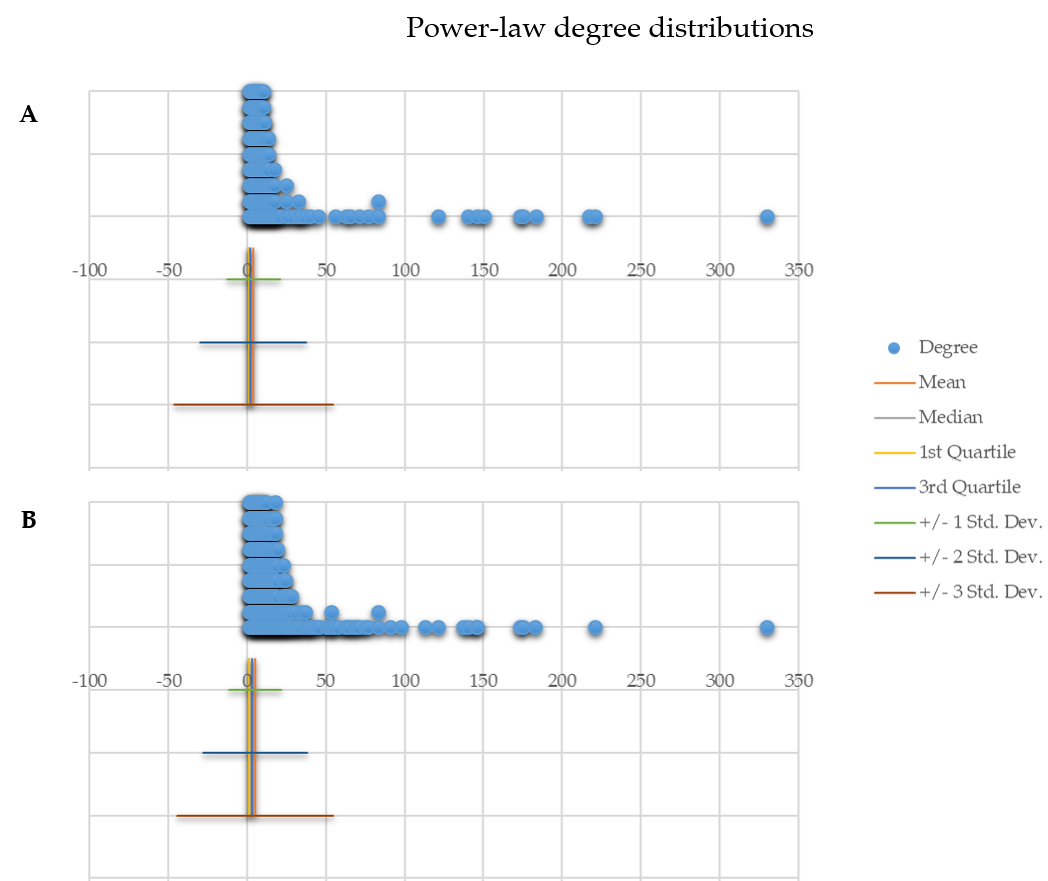


**Figure 1.** The dot scale diagrams show the distribution of nodes degree of connection in (A) brain cortex-specific SALS1 network and (B) brain cortex-specific SALS2 network, where each dot corresponds to a degree value. For the degrees occurring more than once, the dots are placed in columns representing the frequency for that degree value. The graphs remark the coexistence of few hubs with a large number of connections and the most part of the nodes with few links in both the degree distributions.

**Table 2.** Cortex-specific network properties in SALS1 and SALS2 subtypes. The table shows the number of object networks (nodes); number of interactions between pairs of nodes (edges); number of genes used as input gene lists (seeds); highest degree (HD); highest betweenness (HB).

| **Brain cortex-specific PPI network** | **Nodes** | **Edges** | **Seeds** | **HD** | **HB** |
| --- | --- | --- | --- | --- | --- |
| SALS1  SALS2 | 1479  1711 | 2898  4306 | 55  122 | 330  330 | 303998.5  339224 |

**Table 3.** Topological analysis of cortex-specific networks. The table shows the degree mean, standard deviation and cut-off values that were used to classify hub- and non hub-bottlenecks among the total distribution of nodes, as described in methods section. In particular, the latter category were identified among nodes having at least 2 connections with hub-bottlenecks.

| **Brain cortex-specific PPI network** | **Degree mean** | **Standard Deviation** | **Cut-off (mean+2*S.D.)** | **Hub-bottlenecks** | **Non hub-bottlenecks** |
| --- | --- | --- | --- | --- | --- |
| SALS1 | 3.9 | 16.8 | 37.6 | 19 | 338 |
| SALS2 | 5 | 16.5 | 38.1 | 35 | 441 |


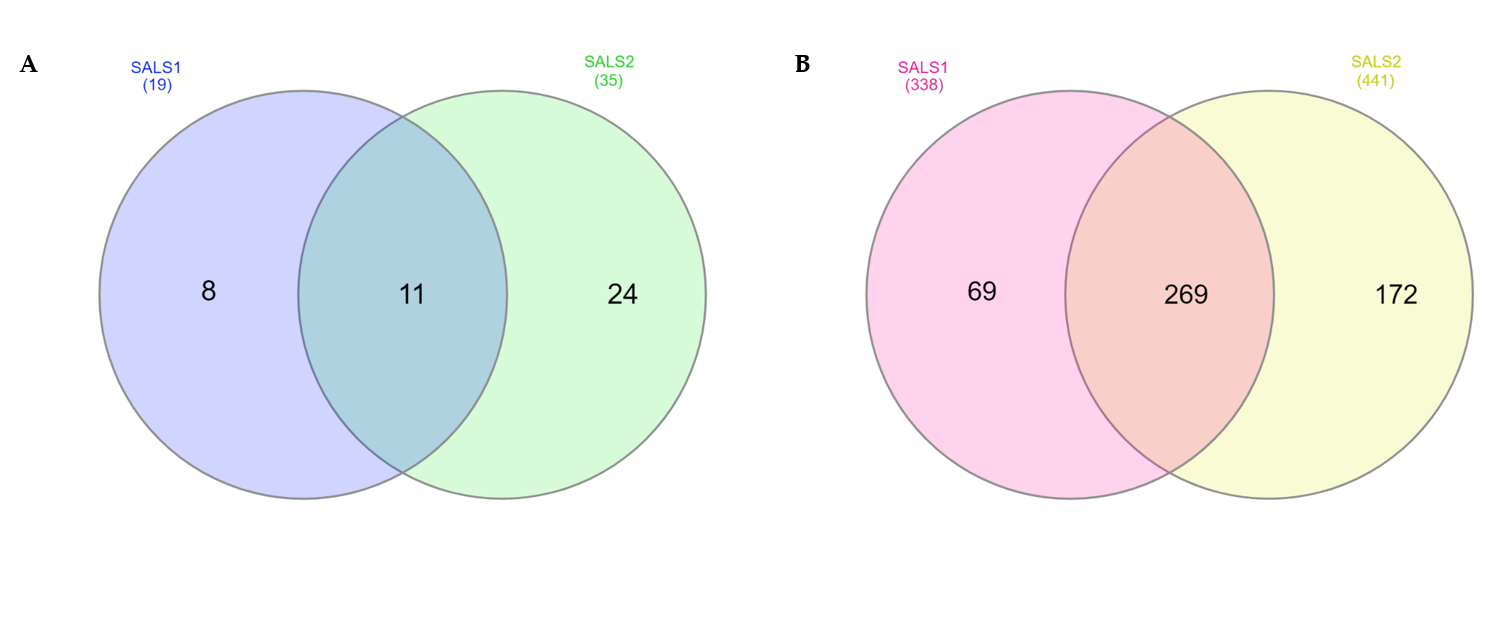


**Figure 2.**  Venn diagrams illustrate the number of hub-bottlenecks (A) and non hub-bottlenecks (B) specific for SALS1 and SALS2 in brain cortex, as well as those falling in their intersections.

**Table 4.** Brain cortex-specific hub-bottlenecks. The table lists 8 hub-bottlenecks specific for SALS1 cluster, 24 for SALS2 cluster, and 11 common to both.

| **Cortex-specific** **hub-bottlenecks** | | |
| --- | --- | --- |
| **SALS1** | **SALS1 ∩ SALS2** | **SALS2** |
| DHX15 | DHX9 | C1QBP |
| EIF4A3 | HNRNPA1 | DDX17 |
| FUS | HNRNPC | DDX5 |
| HNRNPA2B1 | HNRNPD | FN1 |
| HNRNPU | HNRNPK | HNRNPA3 |
| PRPF8 | HSPA8 | HNRNPH1 |
| RBPMS | PCBP1 | HNRNPM |
| SNRPN | SNW1 | HNRNPUL1 |
|  | SRRM2 | KHDRBS1 |
|  | TARDBP | MAGOH |
|  | YBX1 | PABPC1 |
|  |  | POLR2A |
|  |  | RBMX |
|  |  | SF3A2 |
|  |  | SF3B1 |
|  |  | SMN1 |
|  |  | SNRNP200 |
|  |  | SNRPA |
|  |  | SNRPD3 |
|  |  | SRPK2 |
|  |  | SYNCRIP |
|  |  | THRAP3 |
|  |  | U2AF2 |
|  |  | UPF1 |

**Table 5.** Brain cortex-specific non hub-bottlenecks. The table lists 69 non hub-bottlenecks specific for SALS1 cluster, 172 for SALS2 cluster, and 269 common to both.

| **Brain cortex-specific non hub-bottlenecks** | | | | | | | | |
| --- | --- | --- | --- | --- | --- | --- | --- | --- |
| **SALS1** | | | **SALS2** | | | | | |
| ABCA1 | NEDD1 | TCEB1 | ABL1 | DHX15 | ISG15 | PPP2R1A | SMNDC1 | TUBA1A |
| ASB2 | NFKBIA | TNFRSF1B | ACIN1 | DICER1 | ITCH | PPP4C | SNIP1 | TUBA1B |
| ATXN1L | NR3C1 | U2AF2 | ALYREF | DOT1L | ITSN2 | PRKAB1 | SNORD42A | TUBB3 |
| C1QBP | PABPC1 | UPF1 | ANXA1 | DROSHA | LENG8 | PRPF6 | SNRNP40 | TUBB4A |
| CEP57 | POGZ | WIBG | APC | EIF2C1 | LMO3 | PRPF8 | SNRNP70 | UCHL1 |
| CNNM3 | PRMT1 | YTHDC1 | APP | EIF4A3 | LNX1 | PUF60 | SNRPA1 | USP10 |
| COL18A1 | RBFOX1 | ZNF581 | ARF6 | EIF4B | LUC7L2 | RBBP6 | SNRPD1 | USP39 |
| DDX17 | RBM17 |  | ARGLU1 | EPB41 | LUZP4 | RBFOX2 | SNRPD2 | WBP11 |
| DDX5 | RBMX |  | ATG12 | FOS | MAP3K1 | RBL1 | SNRPF | WDR77 |
| EGFR | RELA |  | ATM | FUBP1 | MAPK13 | RIPK2 | SNRPG | WHSC1 |
| ENO1 | RIF1 |  | BANF1 | FUS | MAST3 | RIPK3 | SORBS3 | WWOX |
| FBXL18 | RPL12 |  | BUB3 | GATC | MCM7 | RNPS1 | SRPK3 | XRN2 |
| FN1 | RPL18 |  | C10orf28 | GPN1 | MDC1 | RPA2 | SRSF4 | YAP1 |
| GRAP2 | RPL21 |  | C1orf124 | GRB2 | MED9 | RPA3 | SRSF5 | YWHAE |
| GRK5 | RPS14 |  | CASP8 | GSK3B | MEX3C | RPL18A | SRSF9 | YWHAH |
| HCFC1 | RPS5 |  | CDC73 | GSTK1 | MLL2 | RPL23A | STAT3 | ZBTB1 |
| HNRNPA3 | RPS8 |  | CDK9 | GTF2E2 | MPP1 | SART3 | STK24 | ZNF207 |
| HNRNPH1 | SF3A2 |  | CELF1 | HDAC1 | MSH2 | SF3A3 | STK4 |  |
| HNRNPM | SF3B1 |  | CEP250 | HDAC2 | MTUS2 | SF3B14 | TCEA1 |  |
| HNRPDL | SFN |  | CIRBP | HDAC3 | NAA38 | SF3B2 | TCERG1 |  |
| HNRPLL | SNORA27 |  | CLK2 | HDAC5 | NDRG1 | SF3B5 | TCF3 |  |
| HSP90B1 | SNORD102 | | CLK3 | HECW2 | NOP2 | SFPQ | TDRD3 |  |
| KHDRBS1 | SNORD38B | | CNBP | HNRNPA2B1 | PACSIN1 | SGK1 | TIAL1 |  |
| KPNA2 | SNORD55 |  | COPS6 | HNRNPAB | PAIP1 | SHC1 | TNFRSF10D | |
| KRAS | SNRNP200 | | CPSF2 | HNRNPU | PARN | SIN3A | TNFRSF1A | |
| LGALS3BP | SNRPA |  | CREBBP | HSPD1 | PHF6 | SIRT6 | TNNT1 |  |
| MAGOH | SNRPB |  | CRMP1 | IFIT1 | POLD1 | SMAD3 | TOPORS |  |
| MCM5 | SNRPD3 |  | CTNNBL1 | IFIT2 | POLR1E | SMAD5 | TP63 |  |
| MIR3652 | SRSF10 |  | DBC1 | IFIT3 | POT1 | SMC1A | TRA2B |  |
| MVP | SYNCRIP |  | DDX20 | ILK | PPM1G | SMC3 | TRAF1 |  |
| MYH10 | TAF10 |  | DDX46 | INTS6 | PPP1R8 | SMG1 | TSG101 |  |

| **Brain cortex-specific non hub-bottlenecks** | | | | | | | | |
| --- | --- | --- | --- | --- | --- | --- | --- | --- |
| **SALS1 ∩ SALS2** | | | | | | | | |
| ACTB | CUL7 | FIP1L1 | KHDRBS2 | PABPC4 | RPL28 | SNORD16 | TUBGCP3 |  |
| ADRB2 | DAP3 | FXR2 | KHDRBS3 | PAN2 | RPL3 | SNORD18A | U2AF1 |  |
| AIRE | DCUN1D1 | G3BP1 | LARP4 | PARK2 | RPL4 | SNORD18B | U2SURP |  |
| AKT1 | DDX21 | GABARAPL1 | LARP7 | PAXIP1 | RPL6 | SNORD18C | UBC |  |
| APBB1 | DDX23 | GABARAPL2 | LGR4 | PCBP2 | RPL7 | SNORD19B | UBE2I |  |
| AQR | DDX3X | GEMIN4 | LMNA | PCNA | RPL7A | SNORD24 | UBL4A |  |
| ARRB1 | DDX42 | GIGYF2 | LYAR | PDLIM7 | RPL8 | SNORD32A | VCAM1 |  |
| ARRB2 | DDX50 | GNL3 | MAP1LC3A | PIN1 | RPS11 | SNORD33 | VCP |  |
| ATXN1 | DDX6 | GTPBP4 | MBNL1 | PITX2 | RPS2 | SNORD34 | VHL |  |
| ATXN2L | DGCR8 | H1FX | MDM2 | PNN | RPS24 | SNORD35A | VIM |  |
| AURKA | DHX30 | H2AFX | MEPCE | PPP2R2B | RPS3 | SNORD36A | XRCC5 |  |
| BAG2 | DNAJA1 | H3F3A | MGMT | PRPF19 | RPS3A | SNORD36B | XRCC6 |  |
| BAG3 | DNAJC9 | H3F3B | MIR1306 | PRPF3 | RPS6 | SNORD58A | XRN1 |  |
| BARD1 | EED | HAUS1 | MIR3064 | PRPF40A | RPS7 | SNORD58B | YWHAG |  |
| BCLAF1 | EEF1A1 | HDAC11 | MIR4745 | PRPF4B | RPS9 | SNORD58C | YWHAQ |  |
| BMI1 | EEF2 | HDAC6 | MIR5047 | PSMA3 | RPSA | SNORD61 | YWHAZ |  |
| BRCA1 | EIF2C2 | HDGF | MIR7-1 | PTBP1 | RUVBL1 | SNORD68 | ZC3H11A |  |
| BTRC | EIF2C4 | HIST1H1C | MOV10 | PTCD3 | SART1 | SNORD73A |  |  |
| CAND1 | EIF3B | HLA-B | MTA2 | RAD21 | SEPT9 | SNORD83B |  |  |
| CCDC8 | EIF3F | HNRNPA0 | MYC | RALY | SF1 | SNRPC |  |  |
| CD4 | EIF3H | HNRNPF | MYH9 | RARA | SF3A1 | SQSTM1 |  |  |
| CD81 | EIF3I | HNRNPH3 | NACA | RBM14 | SF3B3 | SREK1 |  |  |
| CDC5L | EIF4A1 | HNRNPL | NCBP1 | RBM3 | SF3B4 | SRPK1 |  |  |
| CDK2 | EIF4G1 | HNRNPR | NCL | RBM39 | SIRT1 | SRRM1 |  |  |
| CDKN1A | ELAVL1 | HSPA5 | NEDD4 | RBM41 | SIRT7 | SRRT |  |  |
| COMMD3-BMI1 | EMD | HSPB1 | NEDD8 | RBM5 | SMARCAD1 | SRSF1 |  |  |
| COPS5 | EP300 | HSPH1 | NFKB2 | RNF2 | SMN2 | SRSF3 |  |  |
| CPSF6 | EPB41L3 | HUWE1 | NFX1 | RNU86 | SMURF1 | SRSF6 |  |  |
| CSDA | ERG | ICAM1 | NONO | RPA1 | SND1 | SRSF7 |  |  |
| CTNNB1 | ESR1 | IGF2BP2 | NOP58 | RPL10 | SNORA48 | STAU1 |  |  |
| CUL1 | ESR2 | IGSF8 | NOS2 | RPL13 | SNORA6 | SUMO2 |  |  |
| CUL2 | EWSR1 | ILF2 | NPM1 | RPL13A | SNORA62 | SUZ12 |  |  |
| CUL3 | EZH2 | ILF3 | NUFIP2 | RPL15 | SNORA64 | TOP1 |  |  |
| CUL4A | FAM120A | IQCB1 | NXF1 | RPL17 | SNORA67 | TP53 |  |  |
| CUL4B | FBL | ITGA4 | OBSL1 | RPL19 | SNORA70 | TRAF6 |  |  |
| CUL5 | FBXO6 | IVNS1ABP | PA2G4 | RPL26 | SNORD10 | TSR1 |  |  |

**Topological analysis of spinal cord-specific SALS1 and SALS2 networks**

**Table 6.** Descriptive statistics summary concerning degree distributions of spinal cord-specific first-order networks.

| **Descriptive statistics** | **SALS1** | | **SALS2** |
| --- | --- | --- | --- |
| **Mean** | | 2.6 | 2.1 |
| **Median** | | 1 | 1 |
| **Mode** | | 1 | 1 |
| **Minimum** | | 1 | 1 |
| **Maximum** | | 165 | 130 |
| **Range** | | 164 | 129 |
| **Standard Deviation** | | 12.3 | 10.7 |
| **Skewness** | | 10.7 | 11.9 |
| **Count** | | 406 | 148 |


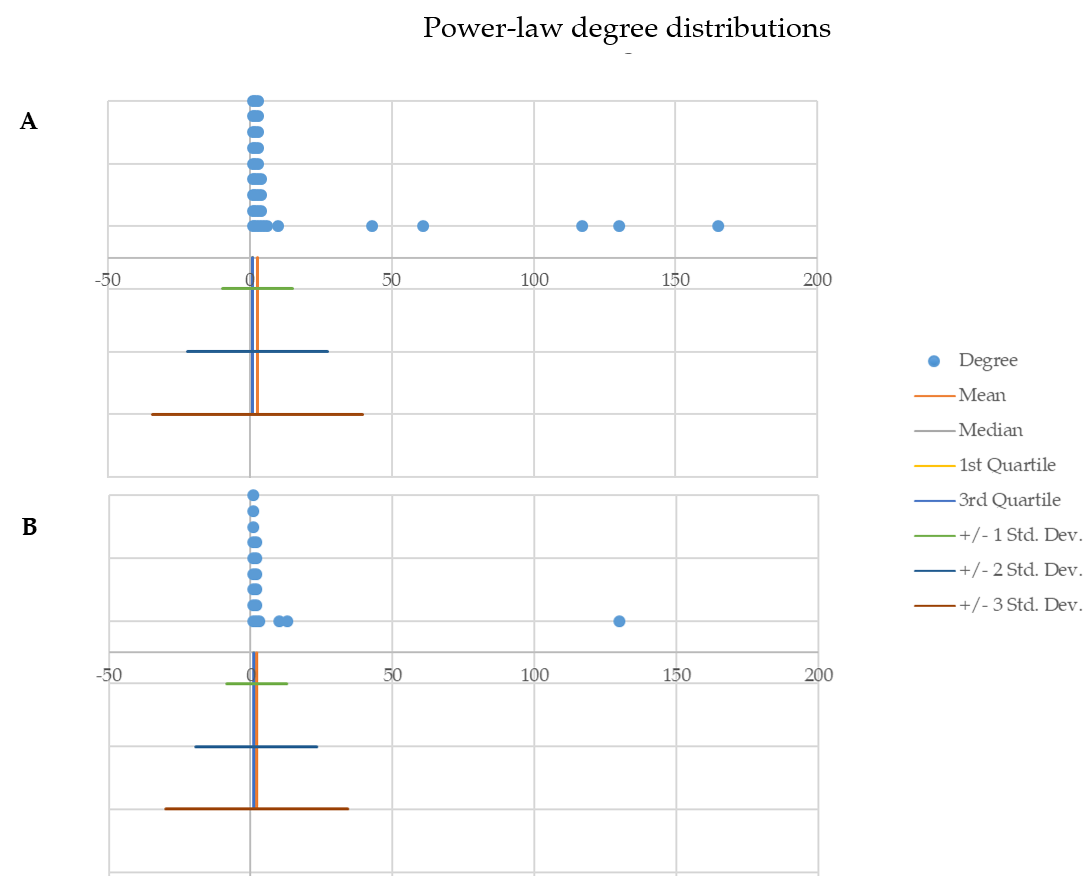


**Figure 3.** The dot scale diagrams show the distribution of nodes degree of connection in (A) spinal cord-specific SALS1 network and (B) spinal cord-specific SALS2 network, where each dot corresponds to a degree value. For the degrees occurring more than once, the dots are placed in columns representing the frequency for that degree value. The graphs remark the coexistence of few hubs with a large number of connections and the most part of the nodes with few links in both the degree distributions.

**Table 7.** Spinal cord-specific network properties in SALS1 and SALS2 subtypes. The table shows the number of object networks (nodes); number of interactions between pairs of nodes (edges); number of genes used as input gene lists (seeds); highest degree (HD); highest betweenness (HB).

| **Spinal cord x-specific PPI network** | **Nodes** | **Edges** | **Seeds** | **HD** | **HB** |
| --- | --- | --- | --- | --- | --- |
| SALS1  SALS2 | 406  148 | 524  153 | 6  3 | 165  130 | 45715.5  10504.9 |

**Table 8.** Topological analysis of spinal cord-specific networks. The table shows the degree mean, standard deviation and cut-off values that were used to classify hub- and non hub-bottlenecks among the total distribution of nodes, as described in methods section. In particular, the latter category were identified among nodes having at least 2 connections with hub-bottlenecks.

| **Spinal cord-specific PPI network** | **Degree mean** | **Standard Deviation** | **Cut-off (mean+2*S.D.)** | **Hub-bottlenecks** | **Non hub-bottlenecks** |
| --- | --- | --- | --- | --- | --- |
| SALS1 | 2.6 | 12.3 | 27.3 | 5 | 92 |
| SALS2 | 2.1 | 10.7 | 23.42 | 1 | 0 |


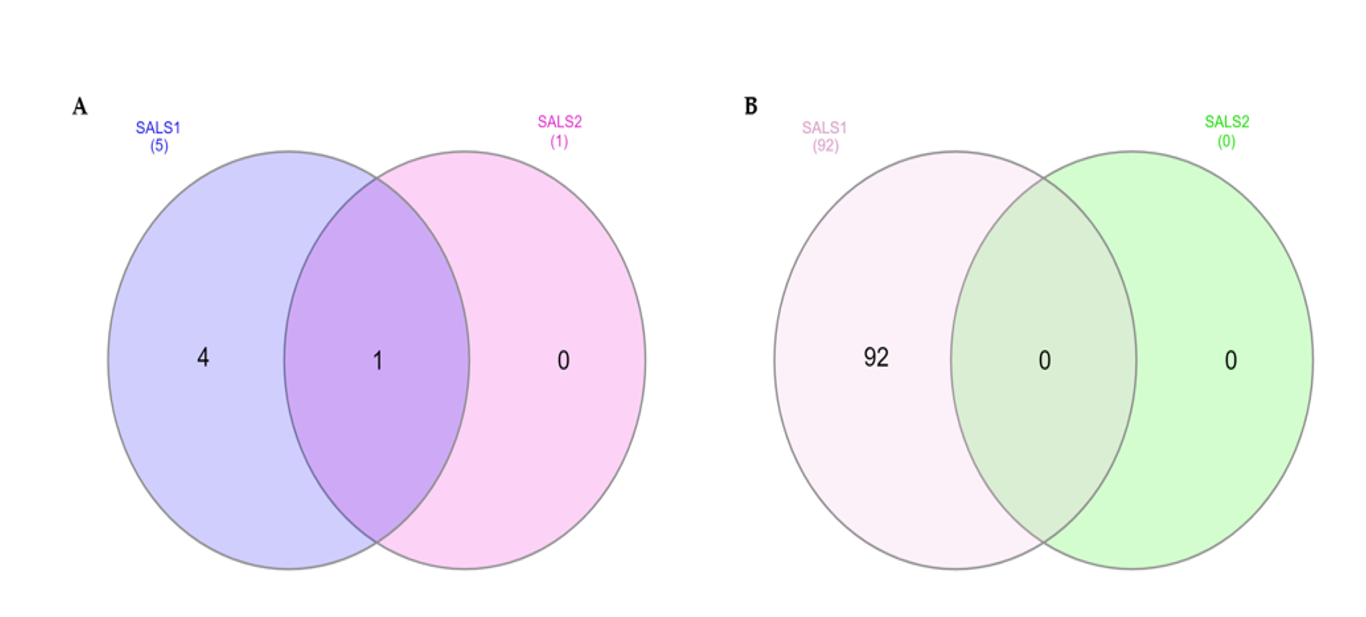


**Figure 4.** Venn diagrams illustrate the number of hub-bottlenecks (A) and non hub-bottlenecks (B) specific for SALS1 and SALS2 in spinal cord, as well as those falling in their intersections where present.

**Table 9**. Spinal cord-specific hub-bottlenecks. The table lists 4 hub-bottlenecks specific for SALS1 cluster, only one common to both, and no one specific for SALS2 cluster.

| **Spinal cord-specific** **hub-bottlenecks** | | |
| --- | --- | --- |
| **SALS1** | **SALS1 ∩ SALS2** | **SALS2** |
| SRRM2 | YBX1 | - |
| PABPC1 |  |  |
| SRRM1 |  |  |
| SF1 |  |  |

**Table 10.** Spinal cord-specific non hub-bottlenecks. The table lists 92 non hub-bottlenecks specific for SALS1 cluster, and no one for SALS2.

| **Spinal cord-specific non hub-bottlenecks** | | | | | | | | |
| --- | --- | --- | --- | --- | --- | --- | --- | --- |
| **SALS1** | | | | | | | **SALS1 ∩ SALS2** | **SALS2** |
| APBB1 | DNAJC8 | GIGYF2 | LMNA | PRPF19 | SIRT7 | YWHAG | - | - |
| CAND1 | EED | GRK5 | MAGOH | PRPF40A | SNORA48 | YWHAZ |  |  |
| CCDC8 | EEF1A1 | HDAC5 | MAPK13 | PTBP1 | SNORA67 |  |  |  |
| CCT5 | EFTUD2 | HNRNPA1 | MIR3064 | RAD21 | SNORD10 |  |  |  |
| CDK2 | EIF2C2 | HNRNPD | MIR4745 | RBM39 | SNRPD2 |  |  |  |
| CLK2 | EIF2C4 | HNRNPF | MIR5047 | RNPS1 | SNW1 |  |  |  |
| CLK3 | EIF4A1 | HNRNPH1 | MIR7-1 | RPS19 | SREK1 |  |  |  |
| CSDA | EIF4A3 | HNRNPK | NFX1 | RPS5 | SRPK1 |  |  |  |
| CUL1 | ELAVL1 | HNRNPL | NONO | SEPT9 | SRSF4 |  |  |  |
| CUL3 | EPB41L3 | ICAM1 | OBSL1 | SF3A2 | SUZ12 |  |  |  |
| CUL7 | ESR1 | IFIT1 | PAN2 | SF3B2 | TP53 |  |  |  |
| DDX21 | EWSR1 | IFIT2 | PAXIP1 | SF3B3 | TRAF6 |  |  |  |
| DDX3X | FBXO6 | IFIT3 | PIN1 | SF3B4 | UPF1 |  |  |  |
| DDX5 | FN1 | IGSF8 | PNN | SFPQ | USP10 |  |  |  |
| DHX16 | FUS | ILF3 | PPP2R2B | SH3KBP1 | XRCC6 |  |  |  |

**Topological role of relevant splicing-related nodes**

**Table 11.** A subset of 6 bottlenecks whose transcripts were deregulated in tissue type pairwise comparisons. Red, green and grey colours reported in table indicate respectively their up, down or no significant deregulation.

| ***Gene*** | ***Motor Cortex*** | | |  | | ***Spinal Cord*** | |
| --- | --- | --- | --- | --- | --- | --- | --- |
|  | ***SALS1 vs Ctrl*** | ***SALS2 vs Ctrl*** |  | | ***SALS1 vs Ctrl*** | | ***SALS2 vs Ctrl*** |
| ***YBX1*** | *Hub bottleneck* | *Hub-bottleneck* |  | | *Hub-bottleneck* | | *Hub-bottleneck* |
| ***HNRNPU*** | *Hub-bottleneck* | *Non Hub-bottleneck* |  | |  | |  |
| ***HNRNPC*** | *Hub-bottleneck* | *Hub-bottleneck* |  | |  | |  |
| ***RBM3*** | *Non Hub-bottleneck* | *Non Hub-bottleneck* |  | |  | |  |
| ***PABPC1*** | *Non Hub-bottleneck* | *Hub-bottleneck* |  | | *Hub-bottleneck* | |  |
| ***DNAJC8*** |  |  |  | | *Non Hub-bottleneck* | |  |
| ***HNRNPA3*** | *Non Hub-bottleneck* | *Hub-bottleneck* |  | |  | |  |
| ***HNRNPUL1*** |  | *Hub-bottleneck* |  | |  | |  |
| ***SNRPN*** | *Hub bottleneck* |  |  | |  | |  |
| ***YTHDC1*** | *Non Hub-bottleneck* |  |  | |  | |  |
