# Supplementary material for: Splicing Players Are Differently Expressed in Sporadic Amyotrophic Lateral Sclerosis Molecular Clusters and Brain Regions
Source: Cells. 2020 Jan 8;9(1):159. doi: 10.3390/cells9010159 (PMC7017305; doi:10.3390/cells9010159)
Supplement: Supplementary file 1 [file cells-09-00159-s001.zip › Supplementary Data S3bis.docx]

**Supplementary Data S3bis - Enriched GOs of DEGs in 7 pairwise comparisons**

Over-represented GOs cellular component enrichment of DEGs listed in S3 for each of the seven pairwise comparisons

**(GO Ontology database Released 2019-10-08)**

**Enriched GOs of DEGs in SALS1 motor cortex vs control cortex**

| **GO cellular component** | **Genes** |  | | | **FDR** |
| --- | --- | --- | --- | --- | --- |
| U4/U6 x U5 tri-snRNP complex | *PRPF6, PRPF8, SNRPN, LSM4* | **4** | / | **31** | 3.39E-04 |
| CRD-mediated mRNA stability complex | *HNRNPU, YBX1, DHX9* | **3** | / | **6** | 1.90E-04 |
| Exon-exon junction complex | *UPF3B, EIF4A3, PNN, SFRS1* | **4** | / | **21** | 9.41E-05 |
| U12-type spliceosomal complex | *SF3B2, DHX15, YBX1, SNRNP35, ZRSR2* | **5** | / | **25** | 3.75E-06 |
| U2-type precatalytic spliceosome | *SF3B2, IK, SRRM2, PRPF6, PRPF8, LSM4* | **6** | / | **50** | 2.84E-06 |
| U2-type catalytic step 2 spliceosome | *SRRM2, PRPF8, BUD31, SYF2, PPIE, SNW1* | **6** | / | **30** | 2.29E-07 |
| U5 SNRP | *PRPF6, PRPF8, CD2BP2, DDX23, SNRPN* | **5** | / | **16** | 6.80E-07 |
| U2-type catalytic step 1 spliceosome | *PRPF8, EIF4A3* | **2** | / | **11** | 4.21E-02 |
| U2AF | *U2AF1, ZRSR2* | **2** | / | **5** | 1.24E-02 |

For each enriched GO, the table shows GO cellular component names, the list of DEGs, the number of DEGs enriched (green) out the total of genes in the GO (red), and the enrichment p-value.

**Enriched GOs of DEGs in SALS2 motor cortex vs control cortex**

| **GO cellular component** | **Genes** |  | | | **FDR** |
| --- | --- | --- | --- | --- | --- |
| CRD-mediated mRNA stability complex | *SYNCRIP, YBX1, DHX9* | **3** | / | **6** | 1.02E-03 |
| mRNA cleavage and polyadenylation specificity factor complex | *CPSF4, FIP1L1, CSTF2T, NUDT21* | **4** | / | **16** | 4.65E-04 |
| supraspliceosomal complex | *ADAR, RBMX, UPF1* | **3** | / | **3** | 2.84E-04 |
| Prp19 complex | *HSPA8, U2AF2, SYF2, POLR2A* | **4** | / | **13** | 2.44E-04 |
| U2-type catalytic step 1 spliceosome | *CASC3, MAGOHB, SNRNP200, RBM8A* | **4** | / | **11** | 1.46E-04 |
| SMN-Sm protein complex | *SNRPD3, STRAP, SMN1, SNRPF, SNRPD2* | **5** | / | **16** | 1.90E-05 |
| U6 snRNP | *LSM3, LSM6, LSM5, LSM7, LSM8* | **5** | / | **8** | 1.38E-06 |
| U5 snRNP | *SNRPD3, CD2BP2, TXNL4A, SNRNP200, DDX23, PRPF18, SNRPD2* | **7** | / | **16** | 1.54E-08 |
| U2-type prespliceosome | *SF3A1, SF3A2, U2AF2, SNRPC, LSM7, SF3B1, LUC7L2* | **8** | / | **18** | 8.35E-10 |
| U2 snRNP | *SF3A1, SF3B5, SF3A2, SNRPD3, HTATSF1, SNRPB2, SF3B1, SNRPD2* | **8** | / | **20** | 1.59E-09 |
| exon-exon junction complex | *CASC3, MAGOH, MAGOHB, UPF1, THRAP3, SAP18, RBM8A, SFRS1* | **8** | / | **21** | 2.07E-09 |
| U1 snRNP | *SNRPA, SNRPD3, SNRPC, SNRNP70, SNRPB2, SNRPF, SNRPD2, LUC7L2* | **8** | / | **20** | 1.55E-09 |
| U12-type spliceosomal complex | *SF3B5, RBM41, SNRPD3, YBX1, LSM7, SNRPF, SF3B1, SNRPD2, PDCD7* | **8** | / | **25** | 6.35E-09 |
| RNA polymerase II, core complex | *POLR2I, POLR2D, POLR2F, POLR2A, POLR2K, POLR2C, POLR2L, POLR2G* | **8** | / | **15** | 2.84E-10 |
| U2-type catalytic step 2 spliceosome | *SRRM2, CWC15, EFTUD2, SNRPD3, SYF2, PPIL1, SNRPB2, SNRPF, PPIE, SNW1, SNRPD2* | **11** | / | **30** | 6.89E-13 |
| U4/U6 x U5 tri-snRNP complex | *LSM3, USP39, LSM6, EFTUD2, SNRPD3, ZMAT2, TXNL4A, SNRNP200, PRPF18, LSM5, LSM7, SNRPF, LSM8, SNRPD2* | **14** | / | **31** | 1.61E-17 |
| U2-type precatalytic spliceosome | *LSM3, SF3A1, SRRM2, SF3B5, LSM6, SF3A2, MAGOHB, EFTUD2, RNF113A, MFAP1, SNRPD3, ZMAT2, TXNL4A, SNRNP200, LSM5, PRPF38A, SNRPB2, LSM7, SNRPF, LSM8, SF3B1, SNRPD2* | **22** | / | **50** | 8.57E-28 |
| pICln-Sm protein complex | *SNRPD3, CLNS1A, SNRPF, SNRPD2* | **4** | / | **6** | 2.68E-05 |
| U2AF | *U2AF2, U2AF1* | **2** | / | **5** | 2.87E-02 |
| histone pre-mRNA 3'end processing complex | *SYNCRIP, YBX1* | **2** | / | **6** | 3.67E-02 |
| U4 snRNP | *SNRPD3, SNRPF, SNRPD2* | **3** | / | **10** | 3.36E-03 |
| SMN complex | *FMR1, STRAP, SMN1* | **3** | / | **10** | 3.31E-03 |
| post-mRNA release spliceosomal complex | *HNRNPM, SNRNP200, SYF2* | **3** | / | **13** | 6.29E-03 |

**Enriched GOs of DEGs in SALS1 and SALS2 spinal cord vs CTRL spinal cord**

| **Supervised cluster** | **GO cellular component** | **Genes** |  | | | **FDR** | |  |
| --- | --- | --- | --- | --- | --- | --- | --- | --- |
| SALS1 | Catalytic step 2 spliceosome | *SRRM1, SRRM2, PABPC1* | 3 | / | 85 | | 6.15E-03 | |
| SALS2 | Spliceosomal complex | *RBMY1B, YBX1, XAB2, TRA2A* | 4 | / | 183 | | 1.11E-04 | |

**Enriched GOs of DEGs in CTRL motor cortex vs spinal cord**

| GO cellular component | Genes |  |  |  | FDR |
| --- | --- | --- | --- | --- | --- |
| U12-type spliceosomal complex | *SNRNP25, YBX1* | 2 | / | 25 | 4.30E-02 |
| catalytic step 2 spliceosome | *HNRNPA1, PABPC1, HNRNPF* | 3 | / | 85 | 9.80E-03 |

**Enriched GOs of DEGs in SALS1 motor cortex vs spinal cord**

| GO cellular component | Genes |  |  |  | FDR |
| --- | --- | --- | --- | --- | --- |
| supraspliceosomal complex | *ADAR, RBMX, UPF1* | 3 | / | 3 | 1.64E-04 |
| CRD-mediated mRNA stability complex | *SYNCRIP, DHX9* | 2 | / | 6 | 2.70E-02 |
| U2 snRNP | *SNRPA1, HTATSF1, SNRPN, SNRPB2, SF3B1, SNRPD2, RBMX2* | 7 | / | 20 | 8.44E-09 |
| U2-type catalytic step 2 spliceosome | *CDC40, SNRPA1, PRPF8, EFTUD2, SYF2, SNRPB2, PPIE, SNW1, SNRPD2* | 9 | / | 30 | 6.47E-11 |
| U1 snRNP | *SNRPC, SNRNP70, SNRPN, SNRPB2, SNRPD2, LUC7L2* | 6 | / | 20 | 3.70E-07 |
| exon-exon junction complex | *UPF3B, CASC3, EIF4A3, UPF1, THRAP3, SAP18* | 6 | / | 21 | 4.60E-07 |
| U4/U6 x U5 tri-snRNP complex | *USP39, PRPF8, EFTUD2, ZMAT2, TXNL4A, PRPF18, SNRPN, SNRPD2, LSM4* | 9 | / | 31 | 8.01E-11 |
| U5 snRNP | *PRPF8, TXNL4A, PRPF18, SNRPN, SNRPD2* | 5 | / | 16 | 5.47E-06 |
| RNA polymerase II, core complex | *POLR2I, POLR2D, POLR2K, POLR2G* | 4 | / | 15 | 1.65E-04 |
| U2-type prespliceosome | *SNRPC, SNRNP70, SNRPN, SF3B1, LUC7L2* | 5 | / | 18 | 8.33E-06 |
| U2-type precatalytic spliceosome | *IK, SNRPA1, PRPF8, EFTUD2, RNF113A, MFAP1, ZMAT2, TXNL4A, SNRPB2, SF3B1, SNRPD2, LSM4, RBMX2* | 13 | / | 50 | 2.80E-15 |
| U2-type catalytic step 1 spliceosome | *CASC3, PRPF8, EIF4A3* | 3 | / | 11 | 2.65E-03 |
| U12-type spliceosomal complex | *SNRNP25, DHX15, SNRNP35, SF3B1, SNRPD2, PDCD7* | 6 | / | 25 | 1.09E-06 |
| post-mRNA release spliceosomal complex | *DHX15, HNRNPM, SYF2* | 3 | / | 13 | 3.74E-03 |
| SMN-Sm protein complex | *STRAP, SMN1, SNRPD2* | 3 | / | 16 | 5.93E-03 |

**Enriched GOs of DEGs in SALS2 motor cortex vs spinal cord**

| GO cellular component | Genes |  |  |  | FDR |
| --- | --- | --- | --- | --- | --- |
| supraspliceosomal complex | *RBMX, UPF1* | 2 | / | 3 | 1.43E-02 |
| CRD-mediated mRNA stability complex | *SYNCRIP, HNRNPU, YBX1, DHX9* | 4 | / | 6 | 2.35E-05 |
| U6 snRNP | *LSM3, LSM6, LSM5, LSM7, LSM8* | 5 | / | 8 | 1.26E-06 |
| RNA polymerase II, core complex | *POLR2I, POLR2D, POLR2F, POLR2J, POLR2K, POLR2C, POLR2L, POLR2G* | 8 | / | 15 | 2.45E-10 |
| exon-exon junction complex | *CASC3, MAGOHB, EIF4A3, PNN, UPF1, THRAP3, SAP18, RBM8A, SFRS1* | 9 | / | 21 | 5.09E-11 |
| U2-type prespliceosome | *SF3A1, SF3A2, U2AF2, SNRPC, SNRNP70, LSM7, SF3B1, LUC7L2* | 8 | / | 18 | 7.23E-10 |
| U2-type precatalytic spliceosome | *LSM3, IK, SF3A1, SRRM2, SF3B5, LSM6, SF3A2, MAGOHB, MFAP1, SNRPD3, ZMAT2, TXNL4A, SNRNP200, BUD13, LSM5, PRPF38A, LSM7, SNRPF, LSM8, SF3B1, SNRPD2* | 21 | / | 50 | 2.89E-26 |
| U2-type catalytic step 1 spliceosome | *CASC3, MAGOHB, EIF4A3, SNRNP200, RBM8A* | 5 | / | 11 | 4.01E-06 |
| U4/U6 x U5 tri-snRNP complex | *LSM3, USP39, LSM6, SNRPD3, ZMAT2, TXNL4A, SNRNP200, PRPF18, LSM5, LSM7, SNRPF, LSM8, SNRPD2* | 13 | / | 31 | 5.64E-16 |
| U2 snRNP | *SF3A1, SF3B5, SF3A2, SNRPD3, HTATSF1, RBMY1E, SF3B1, SNRPD2* | 8 | / | 20 | 1.38E-09 |
| U5 snRNP | *SNRPD3, TXNL4A, SNRNP200, DDX23, PRPF18, SNRPD2* | 6 | / | 16 | 5.55E-07 |
| U2-type catalytic step 2 spliceosome | *SRRM2, CWC15, BUD31, SNRPD3, XAB2, SYF2, SNRPF, PPIE, SNW1, SNRPD2* | 10 | / | 30 | 2.09E-11 |
| U1 snRNP | *SNRPA, SNRPD3, SNRPC, SNRNP70, SNRPF, SNRPD2, LUC7L2* | 7 | / | 20 | 5.18E-08 |
| Prp19 complex | *HSPA8, U2AF2, XAB2, SYF2* | 4 | / | 13 | 2.38E-04 |
| post-mRNA release spliceosomal complex | *HNRNPM, XAB2, SNRNP200, SYF2* | 4 | / | 13 | 2.34E-04 |
| SMN-Sm protein complex | *SNRPD3, STRAP, SMN1, SNRPF, SNRPD2* | 5 | / | 16 | 1.64E-05 |
| U12-type spliceosomal complex | *SF3B5, SNRPD3, YBX1, LSM7, SNRPF, SF3B1, SNRPD2* | 7 | / | 25 | 1.81E-07 |
| mRNA cleavage and polyadenylation specificity factor complex | *CPSF4, FIP1L1, NUDT21* | 3 | / | 16 | 9.86E-03 |
| pICln-Sm protein complex | *SNRPD3, CLNS1A, SNRPF, SNRPD2* | 4 | / | 6 | 2.39E-05 |
| U2AF | *U2AF2, U2AF1* | 2 | / | 5 | 2.82E-02 |
| histone pre-mRNA 3'end processing complex | *SYNCRIP, YBX1* | 2 | / | 6 | 3.65E-02 |
| U4 SnRNP | *SNRPD3, SNRPF, SNRPD2* | 3 | / | 10 | 3.22E-03 |
| SMN complex | *FMR1, STRAP, SMN1* | 3 | / | 10 | 3.18E-03 |
| telomerase holoenzyme complex | *HNRNPU, SNRPD3, HNRNPC* | 3 | / | 20 | 1.70E-02 |

For each GO enriched, the table shows GO cellular component names, the list of genes they belonging to, the number of DEGs enriched (green) out the total of genes belonging to the GO (red), and the enrichment p-value.
